# Identification and Validation of Ifit1 as an Important Innate Immune Bottleneck

**DOI:** 10.1371/journal.pone.0036465

**Published:** 2012-06-20

**Authors:** Jason E. McDermott, Keri B. Vartanian, Hugh Mitchell, Susan L. Stevens, Antonio Sanfilippo, Mary P. Stenzel-Poore

**Affiliations:** 1 Computational Biology and Bioinformatics, Pacific Northwest National Laboratory, Richland, Washington, United States of America; 2 Department of Molecular Microbiology and Immunology, Oregon Health & Sciences University, Portland, Oregon, United States of America; 3 Knowledge Discovery and Informatics, Pacific Northwest National Laboratory, Richland, Washington, United States of America; Tufts School of Medicine, United States of America

## Abstract

The innate immune system plays important roles in a number of disparate processes. Foremost, innate immunity is a first responder to invasion by pathogens and triggers early defensive responses and recruits the adaptive immune system. The innate immune system also responds to endogenous damage signals that arise from tissue injury. Recently it has been found that innate immunity plays an important role in neuroprotection against ischemic stroke through the activation of the primary innate immune receptors, Toll-like receptors (TLRs). Using several large-scale transcriptomic data sets from mouse and mouse macrophage studies we identified targets predicted to be important in controlling innate immune processes initiated by TLR activation. Targets were identified as genes with high betweenness centrality, so-called bottlenecks, in networks inferred from statistical associations between gene expression patterns. A small set of putative bottlenecks were identified in each of the data sets investigated including interferon-stimulated genes (Ifit1, Ifi47, Tgtp and Oasl2) as well as genes uncharacterized in immune responses (Axud1 and Ppp1r15a). We further validated one of these targets, Ifit1, in mouse macrophages by showing that silencing it suppresses induction of predicted downstream genes by lipopolysaccharide (LPS)-mediated TLR4 activation through an unknown direct or indirect mechanism. Our study demonstrates the utility of network analysis for identification of interesting targets related to innate immune function, and highlights that Ifit1 can exert a positive regulatory effect on downstream genes.

## Introduction

Methods of analyzing high-throughput datasets, such as those generated from microarray transcriptomic profiling, are generally targeted at identifying the genes that are most differentially expressed in response to a stimulus. This approach has proven extremely useful for identification of genes considered important for further investigation. However, the important upstream mediators of responses are not always strongly differentially regulated, for example in the case of some of the interferon regulatory factor (IRF) transcription factors that are essential for the innate immune response but induce large downstream effects with only minimal changes in their own expression [1] and so would not be identified by traditional expression analysis approaches. Additionally, traditional analysis considers the behavior of each gene independently from all other genes. A complementary approach that we have developed is to treat multi-stimulus or time point data as a coexpression network and then use the topology of the network to identify points of constriction, or bottlenecks [2], [3], [4], [5], [6]. Bottlenecks are predicted to represent points of control for transitions between system states that are important to the underlying conditions being studied. Though the term bottleneck is used in various ways we here define a functional bottleneck to be a gene whose inactivation causes a measurable effect in the expression of downstream targets, acting either directly or indirectly. Identification of and validation of functional bottlenecks predicted by network analysis should provide insight into the dynamics of the disease-relevant biological processes and their regulation, and potentially serve as targets for clinical intervention.

Neuroprotection against stroke can be induced by preconditioning with Toll-like receptor (TLR) ligands that activate the innate immune system prior to stroke. Preconditioning with systemic administrations of the TLR4 agonist lipopolysaccharide (LPS) or the TLR9 agonist CpG-oligonucleotide (CpG-ODN) provides robust neuroprotection against stroke in mice and nonhuman primates [7], [8], [9]. The responses produced by TLR activation depends on many factors such as the TLR ligand, the cell type, and the environment [10] and these responses set off complex signaling cascade that ultimately affect other cell types and systems. Genomic analysis of the response to preconditioning with LPS, CpG-ODN, or brief ischemia (which is dependent on TLR4) shows that TLR signaling pathway is highly regulated [11]. To identify functional bottlenecks with potential roles in TLR-mediated responses and neuroprotection, we have gathered temporal high-throughput transcriptomic responses in the brain and blood using microarrays that simultaneously evaluate the expression of ∼40,000 genes [11]. By analyzing these large datasets together, it is possible to identify genes of regulatory importance TLR signaling in the system that may be missed by examining a single dataset individually. Additionally, inferred networks provide an abstraction of the system in terms of functional modules that are active at different times and/or under different conditions, which allows placement of bottlenecks in the context of the functional dynamics of the system.

Previously several studies have used computational and experimental approaches to define the regulatory structure of immune cells responding to TLR stimulus and to identify important players in these systems. We have used inferred networks to characterize macrophage response to TLR agonists [3] and neuroprotection in a stroke model [2]. Ramsey, et al. used a large set of microarray experiments and bioinformatics approaches to define functional modules and the regulatory structure of macrophage response to TLR agonists [12]. Amit, *et al.* used a microarray experiments followed by high-throughput siRNA perturbation of a large panel of regulators to define a regulatory network in dendritic cells [13]. Finally, Calvano, *et al.* constructed networks based on the effect of LPS stimulation on leukocytes from human patients [14]. These networks were based on existing knowledge of protein-protein interactions and regulatory relationships and the authors used these networks to identify important subnetworks (pathways) using differential expression overlaid on the network. These and other studies highlight the power of using approaches that employ network analysis that considers the system as a whole as opposed to individual components in isolation. This allows the definition of important components of the immune response, and provides a background for interpretation of the results we present in the current study.

Our goal was to identify topological bottlenecks genes that are involved in the innate immune response. We utilized inferred networks derived from transcriptional data from three different sources, and combined the results to identify candidate bottleneck genes that might play more important and/or universal roles in related TLR-mediated neuroprotection and innate immune processes. The first two sources are blood and brain genomic responses from a study of neuroprotection against stroke in mice using the TLR ligands, LPS, CpG-ODN, or brief middle cerebral artery occlusion (MCAO) to precondition [11]. The brain dataset has been previously described [11] and the blood dataset is described for the first time here. The third source is from a large compendium study of innate immune response in mouse macrophages [12]. The first two datasets examine innate immune responses induced by TLRs in the context of preconditioning-induced neuroprotection against stroke. The third dataset provides an isolated view of innate immune responses induced in macrophages by the administration of TLR ligands. Our hypothesis is that network analysis of transcriptional data from several systems responding to stimulation of TLR-mediated response will allow identification of key effectors of system function, in this case innate immune processes.

Our computational analysis identified bottleneck genes for each dataset analyzed and determined major functional pathways that may be affected by these genes. When comparing all three datasets, we found only six conserved bottlenecks including Ifi47, Axud1, Ppp1r15a, Tgtp, Ifit1, and Oasl2. Ifit1 was further investigated by examining its conserved network neighborhood, which had several overlapping genes in each dataset. Finally, we validated the role of Ifit1 as a functional bottleneck in macrophages by showing that blocking expression of Ifit1 using siRNA dramatically reduced expression of the predicted first-order network genes Usp18 and M×1. This data demonstrates that Ifit1 exerts a regulatory influence over important downstream immune genes when stimulated by LPS, though the mechanism of its action remains unclear. Using our novel approach, network construction using transcriptional data from multiple time course studies and identification of key components using topological analysis, we define six potential key modulators of innate immunity that may also contribute to the neuroprotective response produced by preconditioning.

## Methods

### Datasets used in Computational Analyses

#### Mouse Neuroprotection Studies

Microarray data were obtained from a transcriptional study of a mouse model of neuroprotection during stroke [11]. The datasets used for the computational analyses are the brain and accompanying blood samples from previously published experiments [11]. In brief, groups of C57BL/6 mice (n = 4/treatment/time) received either preconditioning alone, preconditioning plus injurious ischemia (45 min middle cerebral artery occlusion (MCAO)), or injurious ischemia alone. Preconditioning paradigms included: LPS (0.2 mg/kg; i.p.), CpG (0.8 mg/kg; i.p.), saline (i.p.), short-term MCAO (12 min), or sham surgery (12 min). For groups receiving preconditioning alone, mice were euthanized at 3, 24 or 72 hr post preconditioning. In groups receiving preconditioning plus injurious ischemia, MCAO was performed 72 hr following the preconditioning stimulus and mice were euthanized at either 3 or 24 hr post occlusion. Six untreated mice were included as a baseline control group. RNA was isolated from the brain and blood of individual animals. Microarray assays were performed in the Affymetrix Microarray Core of the Oregon Health & Science University Gene Microarray Shared Resource. Labeled cRNA target was quality-checked based on yield and size distribution. Quality-tested samples were hybridized to the MOE430 2.0 array. The array image was processed with Affymetrix GeneChip Operating Software (GCOS). The original.CEL files have been deposited in the Gene Expression Omnibus under the accession number GSE32529. Data was normalized within tissue type (i.e. blood and brain normalized separately) using the Robust Multichip Average method (RMA) [15]. The normalized data was then analyzed using a two-way ANOVA model for each gene, using conditions (LPS, CpG, brief ischemia, saline and sham) and time (3 h, 24 h, 72 h, 3 h post-ischemic event, and 24 h post-ischemic event) as groups, treating the blood and brain datasets independently. Each preconditioning treatment maps to 5 time-points with 3 of those times being prior to the ischemic event and the last 2 occurring post ischemic event. Post hoc comparisons were made using the untreated mice as a control group. P-values were adjusted for multiple comparisons using the method of Hochberg and Benjamini [16]. Genes were identified as significantly regulated if the adjusted p-value was less than 0.05 and the fold change in regulation was greater than or equal to 2.0 compared with unhandled control mice. A total of 7352 and 8388 differentially regulated probesets were identified in the blood and brain analyses respectively and were used for the network inference below.

#### Mouse macrophage data

A large compendium of data from mouse macrophages treated with various innate immune agonists, and with various genetic deletions of important transcription factors and signal transduction pathway members [12] was used for the third dataset. This dataset includes multiple time course studies using 15 different innate immune agonist treatments (including LPS and CpG) and 9 different genetic deletions (transcription factors and signal transduction components). The Affymetrix MOE430 2.0 array was also used in this study and samples processed as described in [12] using RMA and ANOVA with adjusted p-values (p<0.05) and fold change (>2.0) used for significance filtering. This resulted in 6088 differentially expressed probesets for the macrophage that were used as input for network inference below.

### Network Inference

To determine high-dimensional relationships between genes in transcriptomic data we used an approach to infer coexpression networks. For the purposes of network inference we treated each probeset from the microarray analysis as an independent entity, rather than combining expression levels from probesets that represent the same gene. This means that in some cases multiple nodes in the network can correspond to a single gene. For purposes of topological properties, this choice allows determination of bottlenecks without the added level of uncertainty that can be introduced by either combining expression values from different probesets or choosing one probeset as representative of the behavior of a gene. We used an algorithm called context likelihood of relatedness (CLR), which determines similarity between gene expression profiles based on mutual information between the profiles, and then scored the relationships using a Z-score [17]. Though the CLR method was developed to infer regulatory relationships between transcriptional regulators and their targets, we use it here as a method for inferring more general relationships between genes in the form of coexpression networks. For each network we used default parameters for inference using 10 bins for binning data and 3 splines for curve fitting (see [17] for details).

Thresholds for considering a relationship to be an edge in a network were chosen to balance precision and recall as estimated in Faith, et al. [17]. Though the original estimates were based on an examination of a prokaryotic regulatory network, this Z-score threshold is reasonably conservative for determining co-expression networks in our eukaryotic networks. The brain and macrophage networks were accordingly filtered with a CLR Z-score of 5.0 (i.e. only edges with a score of 5 or above were retained). To generate a network with approximately the same number of nodes the blood network was filtered with a CLR Z-score of 6.0. Though the Z-score is a property of the gene-to-gene relationships in the network (as opposed to a property of the individual genes), increasing the Z-score threshold in the network increases the number of genes with no edges in the network, and these are not considered as part of the new network. Keeping the number of nodes similar in all networks was important to allow bottlenecks to be more fairly compared between networks.

The full networks for each of the datasets are provided in a single XGMML-format file that can be opened with Cytoscape [18] Supplemental File S1.

### Topological Identification of Bottlenecks

To identify potential points of constriction for information flow in the inferred networks we analyzed each network topologically. The betweenness centrality topological measure identifies bottlenecks that are predicted to be important to the system [3], [4], [6], [19]. Betweenness is a centrality measure calculated as the percentage of shortest paths between all genes in the network pass through the gene in question, and so is dependent on the global structure of the inferred network. We used custom scripts in the R statistical language [20], using the igraph library [21] and available on request, to calculate topology of the networks. Nodes in each network were ranked according to their network betweenness scores, such that the top bottlenecks were at the top of each network node list. Probes that were in the top 20% ranked by betweenness were considered to be bottlenecks [6], [22].

### Functional Condensation of Networks to Highlight Bottlenecks

To determine clusters for the purpose of network summarization and functional exploration we employed the Louvain method for optimizing the modularity of clusters (communities) identified from a network [23]. This approach works by first optimizing modularity locally in the network, then aggregates nodes from the same cluster and builds a network in which the nodes are these clusters. The result is a network partition that provides clusters with modularity that is close to optimal globally. For each bottleneck that was present in a given network, cluster membership of all neighboring genes (including the bottleneck itself) was identified. All clusters with a membership of 10 probes or more were analyzed for functionality using gene ontology (GO) analysis. In this way, each bottleneck was associated with functional clusters to which it was directly linked in the network. These relationships were visualized using the Cytoscape graph visualization package [18], in which the relationships between bottlenecks and functional clusters could be displayed in one condensed graph. All three branches of GO categorization were used to characterize the clusters.

### Cell Culture and siRNA

RAW 264.7 macrophage cells (obtained from ATCC) were cultured in high glucose DMEM (Gibco) containing 10% fetal bovine serum (Hyclone). Cells were sustained using standard tissue culture techniques in an incubator maintained at 5% CO_2_ and at 37°C. RAW 264.7 cells were plated at ∼30,000 cells/cm^2^ in 6-well plates for 24 hrs. RAW 264.7 cells were transfected with 100 nM Stealth Ifit1 siRNA #58 (Invitrogen; MSS205258) or 100 nM LoGC containing Stealth Negative siRNA (Invitrogen; 10620312) using lipofectamine RNAiMAX (Invitrogen) in Opti-MEM (Gibco) media for 4 hrs. At 24 hr post transfection, RAW 264.7 cells were treated with 1 ng/ml LPS (Sigma) or saline for 3 hr followed by RNA isolation.

### RNA Isolation, Reverse Transcription, and qtPCR

RNA was isolated from RAW 264.7 cells using an RNAeasy Mini Kit (Qiagen). Reverse transcription was performed on 2 ug RNA using an Ominiscript Reverse Transcription kit (Qiagen). Quantitative PCR (qtPCR) was performed using Taqman Gene Expression Assays (Applied Biosystems) for Ifit1 (Mm00515153_m1), Usp18 (Mm01188805_m1), M×1 (Mm00487796_m1), and β-Actin (Mm00607939_s1) with TaqMan Universal PCR Master mix (Applied Biosystems) on an ABI Prism 7700. Results were normalized to β-Actin expression and analyzed relative to untreated controls. The relative quantification of the gene of interest was determined using the comparative CT method (2^−DDCt^). Data is represented as mean ± SEM. The n is greater than or equal to 3 for each experiment. Statistical analysis was performed using GraphPad Prism5 software. Two-way ANOVA with Bonferroni post hoc test was used. Significance was determined as p<0.05.

## Results and Discussion

### Topological Analysis of Networks Inferred from Disparate Data Sources

To infer confident coexpression relationships between genes we used the context likelihood of relatedness (CLR) method [17]. CLR uses the mutual information between the expression profiles of two genes over all conditions examined to calculate a Z score based on comparison with all mutual information scores for each of the two genes, thus providing a network-based mutual information score. Though it was originally designed to infer direct relationships between transcriptional regulators and their targets, we use it here to infer more general coexpression relationships between genes [6], [24]. The resulting networks provide an abstract representation of the states of the system, in which groups of coexpressed genes are linked to each other through coexpression relationships. Supplemental Figure S1 illustrates this by showing how expression dynamics relate to the network structure.

We applied CLR to each dataset independently to generate a matrix of probeset-to-probeset coexpression relationships. To identify bottlenecks we ranked all genes in the network by their betweenness centrality and considered the top 20% to be predicted bottlenecks. We chose to examine the top 20% of nodes based on previous studies [2], [6], [22]. Betweenness centrality is calculated as the fraction of shortest paths between all pairs of nodes in a network that pass through a particular node. Therefore, nodes with high betweenness are points of constriction (bottlenecks) in the network. In coexpression networks the structure of the network reflects temporal and/or functional progression from one state to another [19]. We chose to treat probesets as independent entities in this analysis, rather than choosing one probeset to represent the behavior of a gene for those cases where more than one probeset matches a single gene. This choice means that multiple nodes in the network could represent the same gene.

### Context of Bottlenecks in Inferred Networks

**Figure 1:**
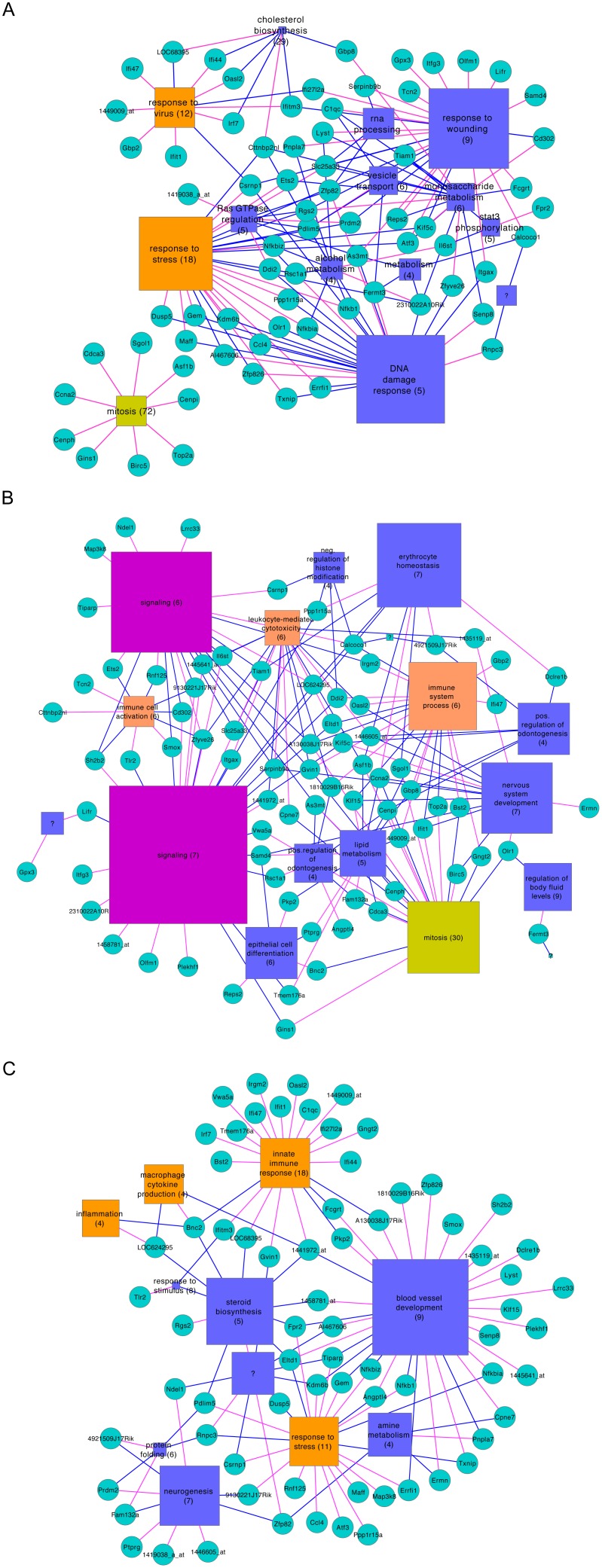
Condensed networks of bottlenecks and functional clusters. A network was inferred from the macrophage innate immune compendium (A), or the blood- (B) or brain-(C) derived transcriptome from the stroke study. Bottlenecks (circles) were identified based on topological betweenness and clusters (squares) were assessed for statistical enrichment in gene ontology functional categories versus genes in the rest of network using the hypergeometric test. Shared functions are indicated by cluster color: orange, immune related/stress response; pink, signaling; green, cell cycle/mitosis. Clusters are labeled with most enriched functional category followed by the negative exponent of the p-value for enrichment. Edges are colored red to indicate that the bottleneck is a member of the cluster that it links to, and red to indicate that the bottleneck is linked to the cluster. Note that not all bottlenecks, clusters or relationships between the two are present in this representation (see text).

We generated condensed graphical representations of the three networks (macrophage, brain, and blood) that depict the interaction of prominent bottlenecks (circles) with network clusters (squares) determined using the Louvain [23] community-finding algorithm (Figure 1). The size of cluster nodes is proportional to the number of genes residing in that cluster, the functional label assigned to each cluster are indicated followed by the negative log of the p-value for enrichment in that function (higher numbers are more significant). Cluster colors (see Fig. 1 legend) indicate general functional groups that are shared between the three networks. It should be noted that not all genes are naturally grouped into large clusters, thus not all nodes and edges of the network are represented. The resulting absence of some connections make some bottlenecks appear to be “dead ends” instead of linking different regions of the graph, as they do in the complete network. In addition, since cluster labels were assigned based on the most prominent ontology grouping (smallest p-value in the hypergeometric enrichment test) associated with each group of genes, assigned labels should be viewed as approximate; other functional characterizations may be applicable. We chose to represent the interactions of genes that were characterized as bottlenecks in at least two of the three networks.

### Conserved Bottlenecks Between Networks

We have previously used the overlap of bottlenecks from disparate networks to identify conserved bottlenecks [25]. Here we identified overlapping bottlenecks from each of the networks and show that there are six bottlenecks in common across all networks (Table 1). One concern with this analysis is that multiple probesets might exist for bottleneck genes, calling in to question their role as functional bottlenecks. However, we note that all of these shared bottlenecks are represented by one differentially regulated probeset in these datasets. This amount of overlap between the three sets is unlikely to occur by chance; the associated p-value is 3e−4 relative to randomly chosen gene sets. Two of these genes, Ifi47 and Tgtp, are members of a family of interferon-induced GTPases that play important roles in response to various pathogens [26]. Two other shared bottlenecks, Ifit1 and Oasl2, are also interferon induced and involved in response to pathogens [3], [27] and were identified as being induced following stroke in preconditioned animals [11]. Previously, we identified Ifit1 as a member of a macrophage ‘core response module’ that was commonly differentially expressed in response to multiple stimulatory signals [3]. The two remaining shared bottlenecks are not known to be interferon induced. Axud1 is an anti-apoptotic factor that suppresses proliferation [28]. Ppp1r15a, also known as Gadd34, is expressed in the ischemic brain and reverses protein synthesis shutdown [29], and can inhibit viral replication [30]. Thus, we postulate that these conserved bottlenecks are important control points for innate immune response. The network neighborhoods of the four interferon-stimulated conserved bottlenecks are shown in Supplemental Figure S2.

**Table 1:** Shared bottlenecks between three inferred networks.

Symbol	ProbeID	Description
Ifi47	1417292_at	interferon gamma inducible protein 47
Axud1	1434350_at	AXIN1 up-regulated 1
Ppp1r15a	1448325_at	protein phosphatase 1, regulatory (inhibitor) subunit 15A
Tgtp	1449009_at	T-cell specific GTPase
Ifit1	1450783_at	interferon-induced protein with tetratricopeptide repeats 1
Oasl2	1453196_a_at	2′-5′ oligoadenylate synthetase-like 2

### Functional Characterization of Ifit1

We had previously identified Ifit1 as a member of the macrophage core response module [3]. Thus, we selected Ifit1 for further investigation. We examined the network context of Ifit1 in the three networks we had inferred. The first-order network surrounding Ifit1 (i.e. all of its direct neighbors) was calculated and the overlapping set of neighbors is listed in Table 2. Because Ifit1 has few neighbors in the blood network, the overlap here was small. However, two genes, Igtp and Usp18, were shared neighbors in all the networks examined. Additionally, Ifi47, already identified as a shared bottleneck, was found to be a shared neighbor of Ifit1 if the blood network neighborhood was extended out one link (i.e. to a second-order network of Ifit1). A number of other genes were shared neighbors in two of the three networks, including many interferon-stimulated genes. We provide the shared neighborhoods of the other conserved bottlenecks as Supplemental File S2.

**Table 2:** Shared neighbors of Ifit1 in three inferred networks.

			Network Neighborhood^a^		
Symbol	ProbeID	Description	Brain	Macrophage	Blood
Igtp	1417141_at	interferon gamma induced GTPase	1	1	1
Usp18	1418191_at	ubiquitin specific protease 18	1	1	1
Ifi47	1417292_at	interferon gamma inducible protein 47	1	1	2
Parp9	1416897_at	poly (ADP-ribose) polymerase family; member 9	1	1	
Irf7	1417244_a_at	interferon regulatory factor 7	1		1
Iigp2	1417793_at	interferon inducible GTPase 2	1	1	
Gbp4	1418392_a_at	guanylate nucleotide binding protein 4	1	1	
–-	1418580_at	–-	1	1	
Oasl1	1424339_at	2′-5′ oligoadenylate synthetase-like 1	1	1	
Ifih1	1426276_at	interferon induced with helicase C domain 1	1	1	
–-	1434380_at	Diabetic nephropathy-like protein (Dnr12)	1	1	
LOC209387	1435665_at	Tripartite motif protein 30-like	1	1	
Rsad2	1436058_at	radical S-adenosyl methionine domain containing 2	1	1	
Tgtp	1449009_at	T-cell specific GTPase	1	1	
Ifit3	1449025_at	interferon-induced protein with tetratricopeptide repeats 3	1	1	
Parp14	1451564_at	poly (ADP-ribose) polymerase family; member 14	1	1	
M×1	1451905_a_at	myxovirus (influenza virus) resistance 1	1	1	
Oasl2	1453196_a_at	2′-5′ oligoadenylate synthetase-like 2	1	1	

a Numbers indicate that the indicated gene is in the first order (1) or second order (2) network of Ifit1 for each network.

In a previous study the effects of siRNA knock-downs of 125 regulators had been assessed on a total of 126 target genes after an initial network-based analysis of TLR stimulation in dendritic cells was performed [13]. The study focused only on transcriptional regulators and so does not include direct validation of any of our predicted shared bottlenecks. However, we assessed the regulatory coherence of the six members of the conserved Ifit1 neighborhood (Table 2) that were assayed in the study: Ifit1, Ifit3, Oasl1, Rsad2, Iigp2, and Irf7. We therefore assessed the correlation of expression profiles for each gene in response to the 125 regulator knock-downs inside the neighborhood versus other genes. This analysis revealed the in-group correlation to be 0.76 while the out-of-group correlation was 0.40 (p-value 2e−16 by t test). This is a slight improvement over the mean correlation of the neighbor genes that are not common between the networks (10 genes; in-group correlation 0.70). The profiles of each of the neighbors is shown as Supplemental Figure S3. These results provide validation that common neighbors of Ifit1 are indeed regulated by the same regulators, even when looking in different cell types. These results show that Ifit1 and its neighbors are strongly positively regulated by the Stats 1, 2, and 4, Etv6, E2f5, and Irf8 in dendritic cells.

Examining the context networks described above we found that in the blood network, Ifit1 links three clusters, one that is strongly identified as a group of genes that function in mitosis, and the other two more weakly associated with immunity and nervous system development. The importance of the nervous system-associated cluster is unclear but it may suggest a response to damage signal that originates from the brain during ischemic stroke. The other two associations suggest that Ifit1 may participate in regulating proliferation of immune cells during stroke-related processes. Similarly, the brain network shows that Ifit1 associates with a cluster of genes related to the innate immune response, and the macrophage network shows that Ifit1 associates with viral response genes that overlap with those in the brain cluster. These are similar groups of genes that demonstrate a potential role for Ifit1 in regulating the inflammatory response to innate immune events in all three network types as can be seen in Table 2.

### Ifit1 Controls Response of Downstream Genes to LPS Stimulation

**Figure 2:**
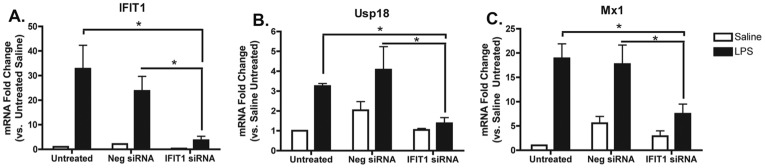
Silencing Ifit1 suppresses LPS activation of Usp18 and M×1. siRNA against Ifit1 or a negative control were introduced into RAW264.7 macrophages by transfections and the macrophages were treated with 1 ng/mL of LPS. Expression of Ifit1 (**A**), Usp18 (**B**) and M×1 (**C**) were measured by RT-PCR at 3 hours post-LPS treatment. The results show that Ifit1 exerts a positive regulatory effect on Usp18 and M×1.

To test the predicted regulatory function of Ifit1, we suppressed Ifit1’s gene expression in RAW 264.7 macrophage cells using siRNA (**Figure 2**). Ifit1 siRNA successfully knocked down baseline levels of Ifit1 and the induction of Ifit1 via the TLR4 ligand, LPS. We next determined the effect of knocking down Ifit1 on the regulation of two genes predicted to be in the Ifit1 first-order network, Usp18 and M×1, in response to LPS. As stated above, Usp18 is one of the few genes that was identified as a neighbor to Ifit1 in all three of our networks, and would therefore be predicted to be regulated by Ifit1. M×1 is an Ifit1 neighbor in the macrophage network and is known to play a critical role in the innate immune response. As predicted from the modeling, when Ifit1 is knocked down both Usp18 and M×1 are significantly suppressed in response to LPS (**Figure 2**). This indicates that Ifit1 is required in the macrophage cell line, for the induction of Usp18 and M×1 in response to LPS-mediated innate immune activation, supporting Ifit1’s role as a functional bottleneck gene, though our results do not distinguish if this is a direct effect, or requires an intermediate factor.

### Conclusions

In this study we used a network-based approach to define important players in cellular networks related to stroke and innate immunity by predicting topological bottlenecks. Bottlenecks, often defined as genes or proteins with a high degree of betweenness centrality in a network, are considered key points of potential biological and functional significance [19], [22], [31]. Previous research in yeast, worm, and fly protein networks demonstrated that proteins with the highest levels of betweenness and centrality were more likely to be evolutionarily conserved and essential to the viability of the system [32]. Thus, we sought to identify bottleneck genes in TLR-mediated innate immune responses in three systems. Two of these systems describe *in vivo* innate immune responses in the setting of TLR preconditioning-induced neuroprotection against stroke in the brain and blood and the third provides an isolated view of innate immune responses in TLR-ligand stimulated macrophage cells *in vitro*. Comparing these three datasets allows us to identify potential bottleneck genes important for innate immune activation.

Previously we have described an approach to compare bottlenecks in networks from proteomics data of HCV-infected cell culture and patient samples and showed that this highlights a small number of conserved bottlenecks between the two systems [25]. This analysis identified a conserved bottleneck involved in fatty acid β-oxidation, DCI, that has been validated as being necessary for HCV replication *in vivo* [5]. In the current study we have used a similar approach in networks inferred from transcriptional data. The number of overlapping bottlenecks between the three networks was found to be highly significant and composed of six genes with some similar functional characteristics. Four of the common bottlenecks are interferon-stimulated genes (ISGs) including Ifi47, Ifit1, Oasl2 and Tgtp and two are members of a family of GTPases known to have roles in response to various pathogens, Ifi47 and Tgtp [26]. These six genes may play critical roles in the regulation of innate immune processes and potentially are involved in promoting neuroprotection against stroke.

Our network analysis showed that the small set of genes predicted to be bottlenecks in each of the networks examined had overlapping neighborhoods, which represent likely targets of regulation. This suggests that the bottleneck genes may be members of functional modules that are conserved in different responses involving innate immune function. For instance, it is interesting to speculate that the four conserved interferon-stimulated bottlenecks may jointly control the regulation of different overlapping aspects of the interferon response, similar to the complex regulation seen in some pathogens [33]. Further, the topological properties of the bottleneck genes indicate that they may drive downstream processes either directly or indirectly, and that the downstream processes should be represented in their network neighborhood. Thus abrogating the expression of a bottleneck gene should have an impact on the expression of some or all of its neighbors. We showed this to be true in the case of Ifit1. When the expression of Ifit1 is suppressed using siRNA in macrophage cells, the expression of downstream genes Usp18 and M×1 were also suppressed in response to LPS. This supports the relationship between Ifit1 and its predicted first-order network, implicating Ifit1 as a functional bottleneck that affects downstream processes.

How Ifit1 exerts its bottleneck functions is currently unclear. A recent study by Pichlmair, *et al.* demonstrated that bone marrow-derived dendritic cells from Ifit1-deficient mice displayed reduced interferon stimulated response element (ISRE) activity when treated with either LPS or CpG; however, type I interferon was not affected [27]. Importantly, the promoter regions of both M×1 and Usp18 contain ISRE sites, thus the suppression of ISRE activation in the Ifit1-deficient mice would likely correlate with a suppression of these genes. This is consistent with our finding that inhibiting Ifit1 using siRNA suppressed Usp18 and M×1 expression in response to LPS. TLRs can directly activate interferon regulatory factors (IRFs) and induce expression of ISRE containing transcripts with out the induction of type I interferons. Thus, Ifit1 may affect the expression of the network genes Usp18 and M×1 by reducing TLR-mediated ISRE activity. Ifit1 may also affect gene expression by interacting with eukaryotic initiation factor 3 (eIF3) to block protein expression [34], [35]. Additionally, Ifit1 interacts with and sequester tri-phosphorylated RNA [33], which are produced during transcription, and the related family member, Ifit2, degrades TNF mRNA [36], although the mechanism has not been identified. Thus, it is possible that Ifit1 may inhibit protein translation or affect mRNA and therefore alter gene expression. The underlying mechanism of Ifit1’s bottleneck function will be examined in further studies.

In conclusion, comparing the topology of networks inferred from three different data sets identified a small set of conserved putative bottleneck genes. We show that our approach using topological analysis of inferred networks from datasets related to TLR-mediated immune response can identify functional bottlenecks that directly or indirectly control the expression of downstream genes, as we demonstrate for Ifit1. Thus, our study shows the utility of analyzing high-throughput data using network approaches to identify potentially significant genes and proteins. The genes identified in this study are now being further investigated to determine their potential functional impact on innate immune processes and neuroprotection against stroke.

## Supporting Information

Figure S1 **Coexpression networks provide an abstraction of expression dynamics in the system.** A portion of the inferred network from the blood transcriptomic data set is shown with circles representing probesets and lines the CLR relationships between them. Each heatmap represents a number of genes located at the indicated point in the network. The time courses for each treatment (LPS, CpG, ischemic preconditioning, saline and sham treatment) are indicated as 3 h, 24 h and 72 h post-treatment (white bar) and 3 h and 24 h post-stroke (pink bar). In the heatmaps, green represents downregulation relative to untreated controls and red represents upregulation. The figure shows that different regions of the network represent different distinct patterns of gene expression, and that no one pattern dominates the network.
(JPG)

Figure S2 **Local networks of conserved IFN-regulated bottleneck genes.** Local networks surrounding conserved bottlenecks in macrophage (A), blood (B) and brain (C) networks are shown for the set of four putative interferon-stimulated conserved bottlenecks (green nodes), Ifi47, Tgtp, Ifit1, and Oasl2. Neighbors of these bottlenecks are colored according to the number of bottlenecks they are neighbors of in any of the networks (tan = 1, yellow = 2, orange = 3, red = 4). This shows that the neighborhood of these genes is largely conserved, and shared in each of the networks, though this is especially evident in the macrophage and brain networks.
(TIF)

Figure S3 **Regulation of the conserved Ifit1 neighborhood in dendritic cells.** RT-PCR expression of target genes included in our Ifit1 neighborhood (rows) are shown against a panel of 125 siRNA knock-downs of regulators (columns) taken from the study by Amit, et al. [13]. In the heatmap, green represents downregulation relative to control siRNA treatment and red represents upregulation. The figure shows that the common Ifit1 neighborhood identified from our three inferred networks is regulated by the same sets of regulators.
(PDF)

File S1 **Cytoscape file containing annotated macrophage, blood and brain networks.**
(TAR)

File S2 **Network neighborhoods of conserved bottlenecks in the macrophage, blood and brain networks.**
(XLSX)
