# Supplementary figures and images for: Identification and Validation of Ifit1 as an Important Innate Immune Bottleneck
Source: PLoS One. 2012 Jun 20;7(6):e36465. doi: 10.1371/journal.pone.0036465 (PMC3380000; doi:10.1371/journal.pone.0036465)

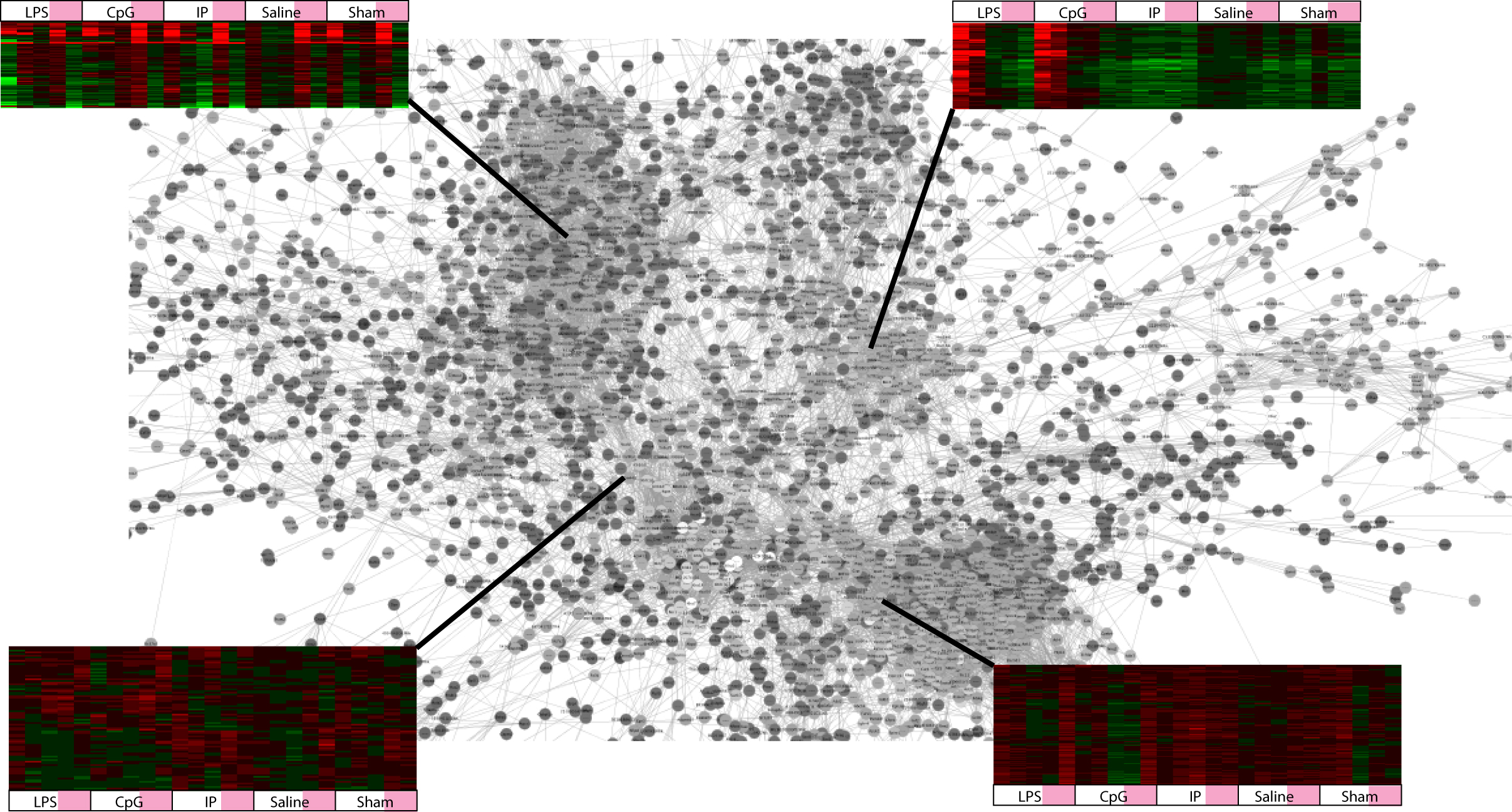

Supplement: Figure S1 — Coexpression networks provide an abstraction of expression dynamics in the system. A portion of the inferred network from the blood transcriptomic data set is shown with circles representing probesets and lines the CLR relationships between them. Each heatmap represents a number of genes located at the indicated point in the network. The time courses for each treatment (LPS, CpG, ischemic preconditioning, saline and sham treatment) are indicated as 3 h, 24 h and 72 h post-treatment (white bar) and 3 h and 24 h post-stroke (pink bar). In the heatmaps, green represents downregulation relative to untreated controls and red represents upregulation. The figure shows that different regions of the network represent different distinct patterns of gene expression, and that no one pattern dominates the network. (JPG) [file pone.0036465.s001.jpg]

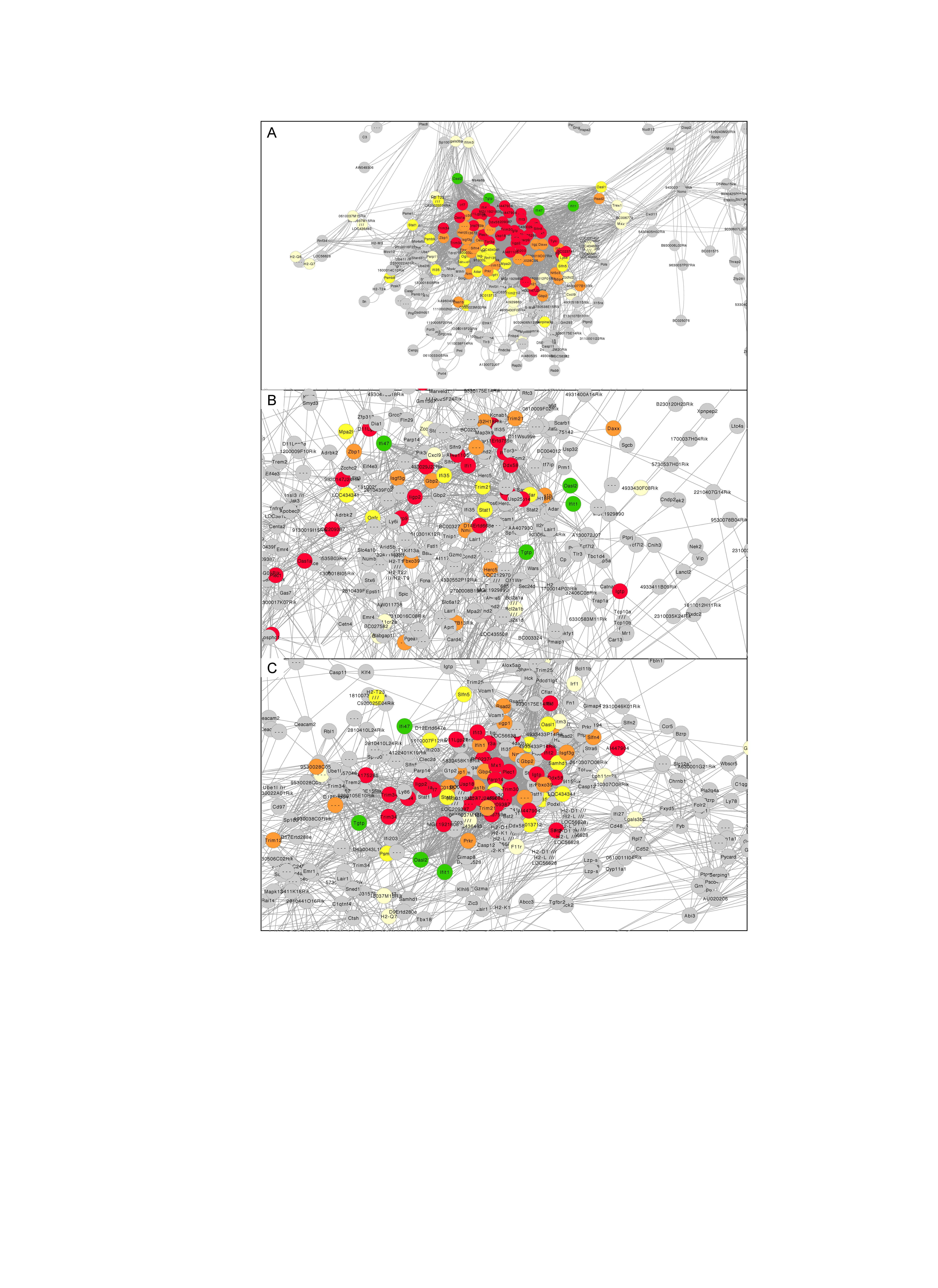

Supplement: Figure S2 — Local networks of conserved IFN-regulated bottleneck genes. Local networks surrounding conserved bottlenecks in macrophage (A), blood (B) and brain (C) networks are shown for the set of four putative interferon-stimulated conserved bottlenecks (green nodes), Ifi47, Tgtp, Ifit1, and Oasl2. Neighbors of these bottlenecks are colored according to the number of bottlenecks they are neighbors of in any of the networks (tan = 1, yellow = 2, orange = 3, red = 4). This shows that the neighborhood of these genes is largely conserved, and shared in each of the networks, though this is especially evident in the macrophage and brain networks. (TIF) [file pone.0036465.s002.tif]

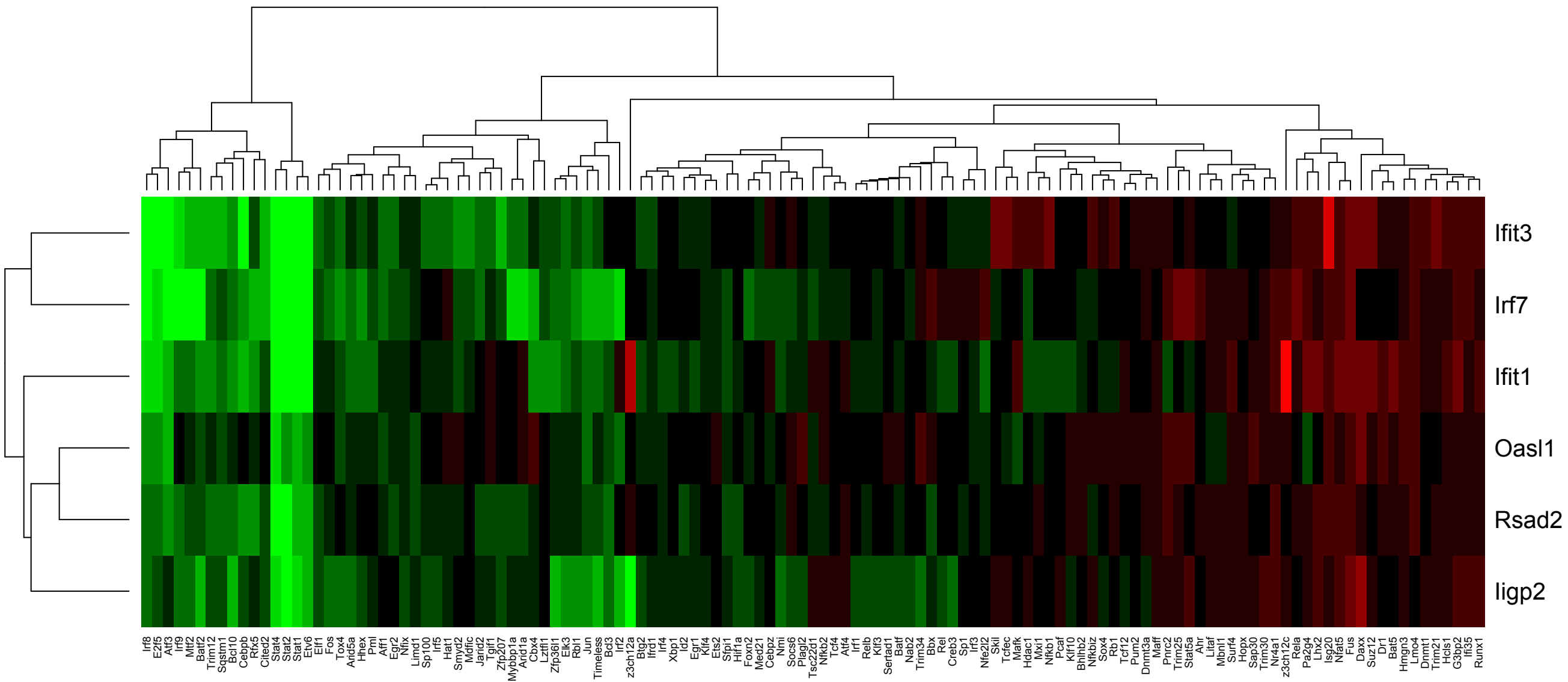

Supplement: Figure S3 — Regulation of the conserved Ifit1 neighborhood in dendritic cells. RT-PCR expression of target genes included in our Ifit1 neighborhood (rows) are shown against a panel of 125 siRNA knock-downs of regulators (columns) taken from the study by Amit, et al. [13]. In the heatmap, green represents downregulation relative to control siRNA treatment and red represents upregulation. The figure shows that the common Ifit1 neighborhood identified from our three inferred networks is regulated by the same sets of regulators. (PDF) [file pone.0036465.s003.pdf]
